# Strengthening the Capacity for Health Promotion: Reflections on Forty Years Since the Ottawa Charter

**DOI:** 10.3389/ijph.2026.1609452

**Published:** 2026-01-28

**Authors:** Verena Biehl, Louise Potvin, Petra Plunger, Anna Wahl, Stephan Van den Broucke

**Affiliations:** 1 Institute of Public Health, ZHAW Zurich University of Applied Sciences, Winterthur, Switzerland; 2 School of Public Health, Université de Montréal, Montreal, QC, Canada; 3 International Union for Health Promotion and Education, Montreal, QC, Canada; 4 Competence Centre Future Health Promotion, Austrian National Public Health Institute (GÖG), Vienna, Austria; 5 Department Society, Health and Health Equity, Austrian National Public Health Institute (GÖG), Vienna, Austria; 6 Psychological Sciences Research Institute, Université Catholique de Louvain, Louvain-la-Neuve, Belgium

**Keywords:** advocacy, capacity building, health promotion, Ottawa Charter, wellbeing societies

Health promotion (HP) is central to public health. The Ottawa Charter [1] marked a milestone by defining health as a dynamic resource for everyday living and initiating a paradigm shift toward empowerment, participation, and creating supportive environments. Over nearly four decades, these principles have shaped public health policies, training programs, and institutional structures. Yet, as the COVID-19 crisis demonstrated, gaps remain that limit HP’s full integration into public health systems. This commentary reviews key achievements of HP since Ottawa and identifies directions for future progress, drawing on the analytic capacity framework proposed by Aluttis et al. [2].

## Leadership and Governance

Over the past 40 years, the HP agenda has been integrated into many national health policies, affecting population health in diverse ways. In Canada and Sweden, HP is a core public health function, while Switzerland, Thailand, Austria, and several Australian states have established independent foundations to fund HP initiatives. In Brazil, HP is embedded in primary care under municipal responsibility. Under WHO leadership, programs such as healthy cities, health-promoting hospitals, schools, and workplaces have supported settings-based and whole-system HP approaches.

However, the COVID-19 pandemic exposed major weaknesses in these integration models. To protect overburdened healthcare systems, many countries redirected HP resources toward emergency public health functions. Health promoters were mobilized for surveillance, hygiene campaigns, and vaccination, while empowerment-oriented HP activities were deprioritized. As a result, vulnerable populations received limited support to cope with the broader social and economic consequences of the pandemic. HP emerged from the crisis weakened, fragmented, and with relevance questioned.

HP leadership also remains weak in many contexts, as legal and political constraints often limit advocacy by national bodies. Implementation of Health in All Policies remains uneven, although local governments show promising practices. At the same time, the planetary health agenda offers new leadership opportunities by emphasizing health co-benefits of environmental sustainability and creating entry points for interministerial collaboration.

## Organizational Structure and Resources

The institutionalization of HP within ministries of health, public health agencies, and local authorities represents a major achievement since 1986 and has clarified mandates for action. Efforts to strengthen HP have included strategic planning, organizational change, policy development, quality systems, accreditation and reward mechanisms, and addressing organizational culture. Despite this progress, the organizational landscape of HP remains highly heterogeneous across countries [3]. Alongside governmental services, a wide range of non-governmental actors operates at national and community levels. While this diversity fosters innovation, insufficient coordination limits the overall potential of HP [4].

A persistent barrier to organizational capacity is inadequate and unstable funding. In most countries, prevention and HP account for about 3% of total health expenditure [5]. Even high-income countries underinvest in HP, while low- and middle-income countries face an even wider gap between HP and curative services. The closure of VicHealth, a well-established independent HP foundation in Victoria, Australia, illustrates how HP structures remain vulnerable to political and fiscal shifts. Strengthening sustainable financing mechanisms is therefore critical to securing HP as a resilient component of public health systems.

## Partnerships and Networks

Over the past four decades, HP networks have expanded considerably, with transnational organizations such as IUHPE, WHO, and EUPHA supporting professional exchange, advocacy, and standard-setting. A key component of these networks is intersectoral collaboration, long recognized as a defining feature of HP and a criterion for effective interventions [6]. Partnerships with non-governmental organizations are crucial for reaching marginalized populations, mobilizing communities, and advocating for structural change. At the policy level, closer alignment with other sectors is increasingly important, particularly in relation to the Sustainable Development Goals (SDGs). Collaboration with the sustainable development sector, through planetary health approaches, enables HP to link health, environmental sustainability, and climate-related co-benefits. Partnerships with the social sector are equally vital for advancing health equity, addressing social determinants of health, and framing health as a human right. While such collaboration requires shared governance and competencies to navigate differing mandates, it offers substantial potential to enhance the societal impact and policy relevance of HP.

Moreover, partnerships and networks play a critical role in strengthening HP education by linking research and practice and facilitating the integration of emerging themes such as climate change, demographic transitions, and digitalization. National initiatives, such as the Austrian Agenda Health Promotion, demonstrate how coordinated networks can foster professionalization and advance HP development across regions.

## Workforce

The HP workforce includes both specialist health promoters and the broader public health workforce applying HP principles in practice [7]. Mainstreaming HP thus requires expanding competencies across multiple professions. Although in some countries HP roles are legally anchored in professional frameworks, training remains insufficiently standardized and uneven across educational programs. In parallel, specialist HP training has expanded. A major driver for this is the IUHPE Core Competency Framework (CompHP), which defines essential competencies for HP specialists and was recently updated [8]. Accreditation of individuals and educational programs linked to this framework has supported professionalization [9].

Nevertheless, the HP workforce continues to face challenges, including unclear professional identity, thematic fragmentation, lack of widely recognized job profiles, and the absence of a shared ethical code. Professional associations increasingly help shape public narratives and influence policy agendas, although stronger coordination could further amplify their impact [4]. These factors collectively contribute to the limited visibility of HP as a distinct profession. Complementary training formats, such as fellowships and short courses, can play a valuable role in building capacity, particularly in regions with less-developed organizational HP structures.

## Knowledge Development

Since 1986, the knowledge base for HP has expanded considerably. University programs, continuing education, and interdisciplinary integration into fields such as social work, education, psychology, and medicine have strengthened conceptual and methodological foundations. Successive WHO conferences and post-Ottawa policy documents have refined principles for action and advanced theory, while handbooks and guidance documents have supported professional standard-setting [3]. Moreover, global and regional conferences, particularly the IUHPE World Conferences, have played a central role in methodological innovation and knowledge exchange. Journals such as Health Promotion International and Global Health Promotion have institutionalized HP as a scientific field and provided platforms for ongoing scholarly debate.

Despite these advances, important evidence gaps persist. Complex, multilevel, and systems-oriented HP interventions remain difficult to evaluate within dominant biomedical research paradigms. Methodological innovation, including realist evaluation, systems approaches, and participatory research, is needed to capture long-term and structural outcomes and inform implementation [10]. Persistent inequities in access to HP training and research capacity, particularly in low- and middle-income countries, further constrain global knowledge development.

Emerging priorities include digital HP, countering misinformation and strengthening digital health literacy; planetary health, integrating sustainability and health co-benefits into practice; equity, decolonization, and Indigenous health; and measurement frameworks capturing systems-level and long-term impacts.

## The Way Forward

Forty years after the Ottawa Charter, HP is inseparable from effective public health practice. The next phase requires sustained, forward-looking investment to ensure that HP continues to contribute to healthier, more equitable, and more sustainable societies. Key priorities include reaffirming the principles of empowerment, participation, and equity; strengthening systemic integration of HP into public health through shared leadership, multisectoral collaboration, and systems learning; enhancing professional identity of HP and visibility through stronger advocacy and clearer career pathways; and advancing future-oriented capacity building that integrates digital, ecological, and equity perspectives to maintain scientific rigor and social relevance.
